# Phosphatidic acid phosphohydrolase modulates glycerolipid synthesis in *Marchantia polymorpha* and is crucial for growth under both nutrient-replete and -deficient conditions

**DOI:** 10.1007/s00425-023-04247-4

**Published:** 2023-10-04

**Authors:** Misao Shimojo, Masashi Nakamura, Ginga Kitaura, Yuta Ihara, Shinsuke Shimizu, Koichi Hori, Masako Iwai, Hiroyuki Ohta, Kimitsune Ishizaki, Mie Shimojima

**Affiliations:** 1 School of Life Science and Technology, Tokyo Institute of Technology, Yokohama, 226-8501 Japan; 2 Graduate School of Science, Kobe University, Kobe, 657-8501 Japan

**Keywords:** ER pathway, Lipid remodeling, Monogalactosyldiacylglycerol, Nitrogen starvation, Phosphate starvation, Phospholipid, Plastid glycolipid

## Abstract

**Main conclusion:**

The phosphatidic acid phosphohydrolase of *Marchantia polymorpha* modulates plastid glycolipid synthesis through the ER pathway and is essential for normal plant development regardless of nutrient availability.

**Abstract:**

Membrane lipid remodeling is one of the strategies plant cells use to secure inorganic phosphate (Pi) for plant growth, but many aspects of the molecular mechanism and its regulation remain unclear. Here we analyzed membrane lipid remodeling using a non-vascular plant, *Marchantia polymorpha*. The lipid composition and fatty acid profile during Pi starvation in *M. polymorpha* revealed a decrease in phospholipids and an increase in both galactolipids and betaine lipids. In *Arabidopsis thaliana*, phosphatidic acid phosphohydrolase (PAH) is involved in phospholipid degradation and is crucial for tolerance to both Pi and nitrogen starvation. We produced two *M. polymorpha PAH* (Mp*PAH*) knockout mutants (Mp*pah-1* and Mp*pah-2*) and found that, unlike Arabidopsis mutants, Mp*pah* impaired plant growth with shorter rhizoids compared with wild-type plants even under nutrient-replete conditions. Mutation of Mp*PAH* did not significantly affect the mole percent of each glycerolipid among total membrane glycerolipids from whole plants under both Pi-replete and Pi-deficient conditions. However, the fatty acid composition of monogalactosyldiacylglycerol indicated that the amount of plastid glycolipids produced through the endoplasmic reticulum pathway was suppressed in Mp*pah* mutants. Phospholipids accumulated in the mutants under N starvation. These results reveal that MpPAH modulates plastid glycolipid synthesis through the endoplasmic reticulum pathway more so than what has been observed for Arabidopsis PAH; moreover, unlike Arabidopsis, MpPAH is crucial for *M. polymorpha* growth regardless of nutrient availability.

**Supplementary Information:**

The online version contains supplementary material available at 10.1007/s00425-023-04247-4.

## Introduction

Inorganic phosphate (Pi) and nitrogen (N) are essential macronutrients for land plants. Thus, when either of these nutrients is deficient, seed plants expand their root surface to improve nutrient uptake from soil and recycle cellular components to maintain metabolic stability. Membrane lipid remodeling is one of the systems plants use to cope with stress induced by Pi limitation and is widely conserved in plants (Nakamura 2013). During remodeling, phospholipids are degraded to release Pi, and part of each resultant glycerol backbone is converted into other glycerolipid species to compensate for any deficiencies in phospholipids in both intracellular membranes and the plasma membrane. In seed plants grown under Pi starvation, phosphatidylcholine (PC) is replaced by digalactosyldiacylglycerol (DGDG) in extraplastidial membranes (Härtel and Benning 2000; Dörmann and Benning 2002), whereas phosphatidylglycerol is replaced by sulfoquinovosyldiacylglycerol (SQDG) in plastids (Yu et al. 2002). The conversion of phosphatidylglycerol to SQDG in plastid membranes is also observed in algae (Iwai et al. 2015). However, in some algae that produce the betaine lipid diacylglyceryl-*N,N,N*-trimethylhomoserine (DGTS), PC is replaced by DGTS under Pi deficiency (–Pi) (Iwai et al. 2015; Cañavate et al. 2017; Oishi et al. 2022). DGTS synthase is widely detected in algae and basal land plants but not in seed plants (Murakami et al. 2018). Like these algae, the common liverwort *Marchantia polymorpha* is one of the non-vascular plants that produce DGTS in membranes (Sato and Kato 1988). Notably, it was recently shown that DGDG, SQDG and DGTS levels in *M. polymorpha* increase during Pi starvation (Hirashima et al. 2021), but the molecular mechanism underlying Pi starvation–induced lipid remodeling has not been analyzed.

In Arabidopsis, phospholipids are degraded to yield diacylglycerols (DAGs) that are provided to plastids for the synthesis of monogalactosyldiacylglycerol (MGDG) and DGDG. DGDG is a non-phosphorus-containing glycerolipid that can compensate for the absence of PC in membranes under Pi starvation. Seed plants have two types of MGDG synthase, namely type A and type B (Awai et al. 2001; Yuzawa et al. 2012). In Arabidopsis, type A MGDG synthase, MGD1, is a crucial enzyme for seedling establishment and biogenesis of photosynthetic membranes (Jarvis et al. 2000; Kobayashi et al. 2007), whereas type B MGDG synthases, MGD2 and MGD3, are not essential for growth under nutrient-replete conditions but are indispensable under Pi starvation (Kobayashi et al. 2009). Expression of *MGD2/3* is enhanced under Pi starvation to upregulate MGDG synthesis for the subsequent production of DGDG (Awai et al. 2001; Kobayashi et al. 2004).

An analysis of membrane glycerolipids in Arabidopsis clarified that phosphatidic acid phosphohydrolase (PAH) is involved in DAG production for galactolipid synthesis during Pi starvation (Nakamura et al. 2009). PAH is classified as an Mg(II)-dependent phosphatidic acid (PA) phosphatase and it contains a haloacid dehalogenase–like a domain that degrades PA to DAG (Han et al. 2006). Arabidopsis PAHs, namely AtPAH1 and AtPAH2, are involved in PC metabolism in the endoplasmic reticulum (ER). The absence of PAHs elevated the content of PC and PA (Nakamura et al. 2009; Eastmond et al. 2010). Growth of the Arabidopsis double knockout mutant *pah1pah2* was not greatly affected under nutrient-replete conditions on a plate (Nakamura et al. 2009), but growth suppression was observed when planted in soil (Eastmond et al. 2010). Under Pi starvation, however, the Arabidopsis double knockout mutant *pah1pah2* significantly reduced the tolerance to Pi starvation (Nakamura et al. 2009). Later, the hypersensitive phenotype of the *pah1pah2* double mutant was also observed during N starvation (Yoshitake et al. 2017). Notably, MGDG turnover in plastids was reduced in mutant *pah* plants, whereas it was enhanced in PAH-overexpressing plants. Thus, it was suggested that DAG synthesized by PAH is provided for plastid glycolipid synthesis through the ER pathway and contributes to the maintenance of plastid membranes under Pi or N starvation (Yoshitake et al. 2017).

PAH is well conserved among eukaryotes and contributes to the various lipid metabolism. In Yeast, PAH1 maintains the phospholipid metabolism during exponential growth and supplies DAG for triacylglycerol synthesis during stationary growth. Indeed, triacylglycerol content in a yeast *pah1* mutant was 92% lower than in wild type (WT) during stationary growth (Carman and Han 2019). It is unrevealed at what point in plant evolution PAH began to be involved in glycolipid synthesis under Pi starvation. As non-vascular plants do not have type B MGDG synthase, we expected to find a clue about the role of PAH in glycolipid synthesis of the common ancestor of land plants.

In this study, we produced *M. polymorpha PAH* (Mp*PAH*) knockout mutants using genome editing and analyzed the growth phenotype and membrane glycerolipid composition compared with WT under Pi or N deficiency (–N). Like Arabidopsis, the *M. polymorpha* mutants exhibited a reduction in plastid glycolipid synthesis through the ER pathway. However, the growth of the *M. polymorpha* mutants was impaired regardless of nutrient availability. Thus, we propose that Mp*PAH* codes a phosphatidic acid phosphohydrolase and MpPAH contributes as same to the ER pathway of glycerolipid synthesis as AtPAH although the pathway might be more crucial for *M. polymorpha* growth compared with that of Arabidopsis.

## Materials and methods

### Plant materials and growth conditions

The *M. polymorpha* strain Tak-1 served as the WT strain in this study. Thalli were maintained asexually with half-strength Gammborg’s B5 medium containing 1% (w/v) agar (hereafter, control medium). For –Pi medium, NaH_2_PO_4_ was excluded from the control medium. For –N medium, KNO_3_ and (NH_4_)_2_SO_4_ were excluded from the control medium and 12.41 mM KCl was supplemented. For each experiment, 40 plants were grown on a cellophane sheet that covered the medium in a square plate (sterilized no. 2, Eiken Chemical Co., LTD., Tokyo, Japan). Each plate was filled with 60 ml of medium. Cultivation conditions were 23 °C and continuous light. For analysis of nutrient starvation, gemmas were cultivated on a control medium for 6 days, transferred to either fresh control medium, –Pi medium, or –N medium, and grown for another 6 days.

### Generation of CRISPR-Cas9 mutants

The target locus of Mp*PAH* for CRISPR/Cas9 genome editing was selected with CasFinder (Aach et al. 2014) and CasOT (Xiao et al. 2014). A CRISPR/Cas9 genome editing vector was constructed according to Sugao et al. (2018). The guide RNA sequences 5’-CTCGGAGACGAGCTAGCCTCGTTA-3’ and 5’-AAACTAACGAGGCTAGCTCGTCTC-3’ were annealed and ligated into pMpGE_En3 that had been digested with BsaI (New England Biolabs Inc., Ipswich, MA, USA). Using the Gateway LR Clonase II (Thermo Fisher Scientific, Waltham, MA, USA), the guide RNA expression cassette was transferred into pMpGE010. Transformation of *M. polymorpha* was mediated by *Agrobacterium tumefaciens* GV3101 (pMP90) carrying a genome editing vector and was performed according to Kubota et al. (2013)

### Lipid extraction

Total lipid was extracted from plant tissue according to the modified Bligh and Dyer method (1959). For whole tissue, a maximum of 1 g fresh weight was frozen in liquid nitrogen. For the first extraction step, frozen thalli were crushed in liquid nitrogen and suspended with 6 ml chloroform:methanol (1:2, v/v). The suspension was centrifuged at 3000×*g* for 5 min and the supernatant was collected. The pellet after centrifugation was rinsed with 1.5 ml chloroform:methanol (1:2, v/v), and centrifuged at 3000×*g* for 5 min. The rinse step was repeated three times. All three rinse solutions (i.e., ~ 4.5 ml) were mixed with the first extracted solution (i.e., ~ 6 ml), 3.5 ml chloroform and 7 ml of 1% (w/v) KCl. After centrifugation at 3000×*g* for 5 min, the organic phase (containing the extracted total lipid) was collected and dried under N_2_ gas at 30 ℃, and then the residue was dissolved in an aliquot of chloroform:methanol (2:1, v/v) and stored at – 20 ℃. For rhizoid lipids, rhizoids were cut off with scissors, frozen in liquid nitrogen, and stored at – 80 ℃. The frozen rhizoids (~ 20 mg) were suspended in 300 µl methanol and pulverized with a Multi-beads Shocker (Yasui Kikai Co., Osaka, Japan). The suspension was mixed with ~ 180 µl of 1% (w/v) KCl and 750 µl chloroform:methanol (1:2, v/v). After centrifugation at 17,700×*g* for 5 min, the supernatant was collected as the first extracted solution. The pellet after centrifugation was rinsed with 150 µl chloroform:methanol (1:2, v/v) and 40 µl of 1% (w/v) KCl, and centrifuged at 17,700×*g* for 5 min. The rinse solution was mixed with the first extracted solution, 300 µl chloroform and 300 µl of 1% (w/v) KCl. After centrifugation at 17,700×*g* for 5 min, the organic phase (containing the extracted total lipid) was collected and dried under N_2_ gas at 30 °C, and then the residue was dissolved in an aliquot of chloroform:methanol (2:1, v/v) and stored at – 20 ℃.

### Lipid analysis

Polar glycerolipids were separated by two-dimensional thin-layer chromatography (TLC). Lipid solution (600 µg total lipid) was spotted on a silica gel plate (105,721, Merck KGaA, Darmstadt, Germany). The first dimension was developed with chloroform/methanol/7 M ammonia (120:80:8, by vol.). After drying for 30 min, the second dimension was developed with chloroform/methanol/acetic acid/ water (170:30:15:3, by vol.). Triacylglycerols were separated by one-dimensional TLC with hexane/diethyl ether/acetic acid (80:20:2, by vol.). Lipid solution (1200 µg total lipid) was spotted on the silica gel plate. After separation, the plate was sprayed with 0.01% (w/v) primuline in 80% aqueous acetone. Lipids were visualized under ultraviolet light. The silica containing the identified lipids was scraped from the plate. An internal standard of heneicosanoic acid (H5149, Merck) was dissolved in chloroform at a concentration of 1 mM. The silica powder was incubated with a mixture of 100 µl of the internal standard, 300 µl of methanol and 300 µl of hydrogen chloride solution 3 M in methanol (90964, Merck) at 85 ℃ for 1 h. Fatty acid methyl esters (FAMEs) were extracted with hexane. Hexane was evaporated at 30 ℃ with N_2_ gas. The resulting dry FAMEs were suspended in 100 µl hexane (Kobayashi et al. 2006; Murakawa et al. 2014). FAMEs were separated on a ULBON HR-SS-10 (length, 25 m; internal diameter, 0.25 mm; Shinwa Chemical Industries Ltd., Kyoto, Japan) and detected by a gas chromatograph (model GC-2030, Shimadzu Co., Kyoto, Japan) equipped with a flame ionization detector. Nitrogen was used as a carrier and make-up gas. The injection volume was 1 µl with a split ratio of 1:50. The injection port and detector temperatures were 250 ℃. The linear velocity of the carrier gas was 13 cm s^−1^. The column temperature program was as follows: temperature was held at 180 ℃ for 26 min, increased to 184 ℃ at 1 ℃ min^−1^, increased to 188 ℃ at 5 ℃ min^−1^, increased to 195 °C at 1 ℃ min^−1^, increased to 200 ℃ at 5 °C min^−1^, increased to 210 ℃ at 3 ℃ min^−1^, and held at 210 ℃ for 2 min. Absolute amounts of FAMEs were calculated using the internal standard C21:0. Calibration curve solutions were made with an FAME mixture (GLC462, Nu-Chek Prep, Inc., Elysian, MN, USA). For PA analysis, liquid chromatography was carried out with an LC-2040C 3D system (Shimadzu) using a Kinetex polar C18 column (2.1 × 150 mm) with a 2.6 µm particle size (Phenomenex Inc., Torrance, CA, USA). Mass spectrometry was carried out with an LCMS-8050 tandem quadrupole mass spectrometer (Shimadzu). The flow rate was 0.2 ml min^–1^. An isocratic program was used, and the solvent was a mixture of 250 ml of 2-propanol, 200 ml of water, 50 ml of acetonitrile, 568 µl of 88% formic acid, and 750 µl of 28–30% ammonia solution (FUJIFILM Wako Pure Chemical Co., Osaka, Japan) containing phosphoric acid (final concentration, 50 µM). The column oven temperature was 40 ℃ and the sample rack temperature inside the autosampler was 5 ℃. Total lipid was diluted to 100 ng µl^–1^ with the solvent, and 5 µl of that diluted sample was used for analysis. Running time for each sample was 80 min. The column effluent was routed directly into the electrospray ionization source of the mass spectrometer. The nebulizing gas flow was 3 l min^−1^, heating gas flow was 10 l min^−1^, and the drying gas flow was 10 l min^–1^. The collision-induced dissociation gas pressure was set to the tuning file created by the manufacturer. The interface temperature was 300 ℃, the desolvent line temperature was 250 ℃, and the block heater temperature was 400 ℃. PA was measured by MRM by setting the ion source to – 3 kV (electrospray ionization negative), and Q1 and Q3 resolution was set to ‘Unit’ (offset value: 5000). Table 1 lists the conditions for measuring each PA molecule.

**Table 1:** The condition for measuring PA. Fatty acids were listed in ascending order of retention time. Shimojo et al.

Fatty acid composition	time range of detection [min]	*m*/*z* of precursor	*m*/*z* of product (1)	*m*/*z* of product (2)	Dwell time [msec]	Collision energy [V]
18:3, 18:3	17–20	691.43	277.22	–	47	35
20:5, 18:3	17–21	715.43	277.22	301.22	47	35
16:3, 16:0	17–23	641.42	249.19	255.23	47	35
20:5, 20:5	17.5–22	739.43	301.22	–	47	35
18:3, 18:2	22–25	693.45	277.22	279.23	47	35
20:5, 18:2	22–26	717.45	279.23	301.22	47	35
20:4, 18:3	22–26	717.45	277.22	303.23	47	35
20:5, 20:4	22–26	741.45	301.22	303.23	47	35
18:3, 16:0	26–32	669.45	255.23	277.22	47	35
20:4, 20:4	27–33	743.47	303.23	–	47	35
20:5, 16:0	27–33	693.45	301.22	255.23	47	35
18:2, 18:2	28–32	695.45	279.23	–	47	35
18:3, 18:1	28–33	695.45	277.22	281.25	47	35
20:4, 16:0	30–40	695.45	303.23	255.23	47	35
18:2, 16:0	33–40	671.47	255.23	279.23	47	35
20:4, 18:1	33–43	721.48	281.25	303.23	47	35
18:1, 16:0	44–55	673.48	255.23	281.25	47	35

For MGDG analysis using the LC–MS/MS system, some parameters were changed from the parameters of PA analysis. Running time for each sample was changed to 43.2 min and ion source of mass spectrometry was set to 4 kV (electrospray ionization positive). Table 2 lists the conditions for measuring each MGDG molecule.

**Table 2:** The condition for measuring MGDG. Fatty acids were listed in ascending order of retention time

Fatty acid composition	time range of detection [min]	*m*/*z* of precursor	*m*/*z* of product (1)	*m*/*z* of product (2)	*m*/*z* of product (3)	Dwell time [msec]	Collision energy [V]
18:3, 16:3	7–14	764.53	567.44	335.28	307.25	97	− 30
18:3, 16:0	23–31	770.58	573.49	335.28	313.30	97	− 30

### Quantitative reverse transcription-PCR

Whole plant tissue (~ 0.4 g) was macerated in liquid nitrogen. The resulting powder was homogenized in a threefold volume of RNA extraction buffer (0.8% (w/v) SDS, 25 mM Tris–HCl pH 7.5, 25 mM KCl, 25 mM MgCl_2_) and a threefold volume of water-saturated phenol. After centrifugation, the aqueous layer was again extracted with a 0.5 volume of chloroform and a 0.5 volume of water-saturated phenol, and this two-phase partitioning was carried out a total of three times. The last aqueous layer was mixed with an equal volume of isopropanol, and after centrifugation the pellet was treated with DNase I, extracted with a 0.5 volume of water-saturated phenol, a 0.5 volume of chloroform, and a 0.1 volume of 3 M sodium acetate. After centrifugation, RNA in the aqueous layer was precipitated by mixing with a 2.5 volume of ethanol.

Reverse transcription of each RNA sample was performed using SuperScriptIII reverse transcriptase (Thermo Fisher Scientific). The resulting cDNA was amplified with TB Green Premix Ex Taq II (Tli RNase H Plus) (Takara Bio Inc., Shiga, Japan), and the signal was quantified with the Thermal Cycler Dice^®^ Real Time System III (TaKaRa Bio Inc.). The Ct value for each target cDNA was normalized with the value obtained for the control gene Mp*APT* (Mp3g25140) (Marcoux et al. 2015). ΔΔCt values were calculated using the corresponding ΔCt value for WT. The primers used were listed in Table 3.

**Table 3:** Primer sequences for qPCR

Gene	Primer name	Primer sequence (5’–3’)
*MpPAH*	MpPAH Fw	TTATTCGAAGGGCTCCACAC
	MpPAH Rv	AGCTCAACTCGTCGGTATCAC
*MpMGD*	MpMGD Fw	ACCAACATGGTAGAGTGGATGG
	MpMGD Rv	CTGGCCAGCAATGTAGCTGT
*MpDGD*	MpDGD Fw	GACCACGCTGATGATACGCT
	MpDGD Rv	TGGTCTGCGCAGACTACAATC
*MpSQD1*	MpSQD1 Fw	GATGGAGTGTTCGGCACTG
	MpSQD1 Rv	CATCTCACCGTATCCCTGATG
*MpSQD2*	MpSQD2 Fw	GAAGGGTCAGCCTGCAACT
	MpSQD2 Rv	CCATCACCAACGATAGCCAA
*MpBTA1*	MpBTA1 Fw	TGTGATCTGGAGAAGCGCAT
	MpBTA1 Rv	TGGCGTACATGTTCACTCG
*MpAPT*	MpAPT Fw	CGAAAGCCCAAGAAGCTACC
	MpAPT Rv	GTACCCCCGGTTGCAATAAG

## Results

### A loss-of-function mutation in Mp*PAH* perturbs growth even under Pi-replete conditions

**Fig. 1:**
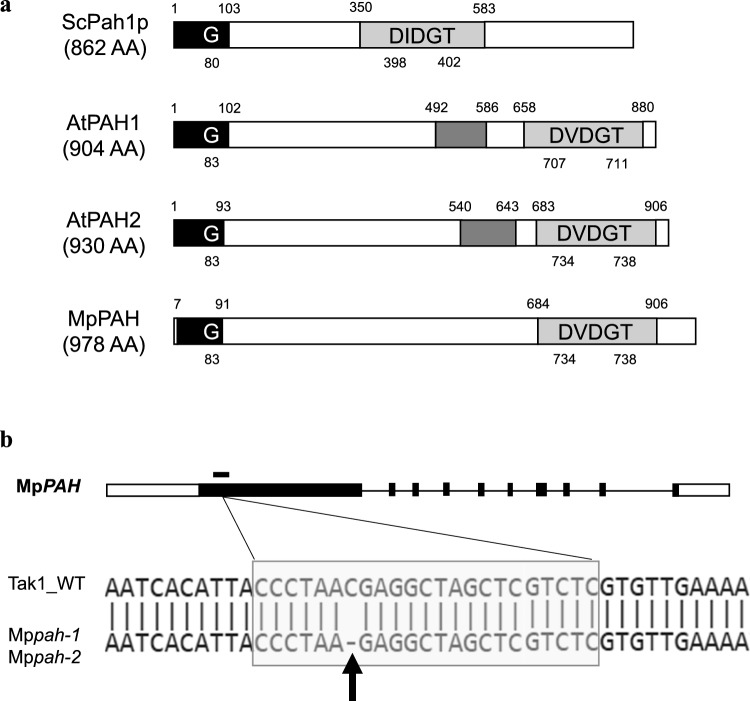
**a** A schematic diagram of PAH proteins. Black, dark gray and gray boxes indicate N-terminal Lipin, Lipin middle and LNS2 domains, respectively. Domains were predicted with InterPro 92.0. The start and end point of the domains are indicated above each construct. Conserved catalytic motifs are shown on each LNS2 domain. The start and end point of the motifs are indicated below each construct. Sc, *Saccharomyces cerevisiae*; AA, amino acids. **b** The single point mutation in Mp*PAH* of each of the two Mp*pah* mutants. Filled boxes correspond to exons of Mp*PAH*. Open boxes correspond to the 5’ and 3’ untranslated regions. The black bar above the first exon and the gray-shaded sequences represent the target sequence of Cas9. The black arrow indicates the single base pair deletion in the two Mp*pah* mutants

To investigate the physiological role of PAH of the liverwort *M. polymorpha* (henceforth denoted as Marchantia), we searched for PAH genes in the genomic sequence database. Marchantia species have simple genomes implying that whole-genome duplication has not occured during the evolution of this lineage (Bowman et al. 2017). Marchantia has one gene, namely Mp3g21770 (Mp*PAH*), that is a homolog of the Arabidopsis *PAH* genes (Rico-Reséndiz et al. 2020). MpPAH is annotated to contain a Lipin/Ned1/Smp2 (LNS2) domain, which is conserved among eukaryotes as a haloacid dehalogenase–like domain (Fig. 1a). The LNS2 domain of MpPAH has the amino acid catalytic motif DVDGT, as does AtPAH (Nakamura et al. 2009; Eastmond et al. 2010; Mietkiewska et al. 2011; Craddock et al. 2015); as such, we considered MpPAH to be a Mg^2+^-dependent phosphatidate phosphatase. The glycine residue in N-terminal Lipin (NLIP) domain which is required for phosphatidate phosphatase activity in yeast PAH1 (Han et al. 2007) was also conserved in AtPAH1, AtPAH2 and MpPAH (Fig. 1a).

In yeast, phosphorylated PAH1 localizes in the cytoplasm. When it is dephosphorylated by Nem1p-Spo7p protein phosphatase complex, it can translocate to the membrane anchoring with an N-terminal amphipathic helix (Karanasios et al. 2010). As neither a transit peptide nor a signal peptide was predicted, MpPAH was considered to localize in the cytoplasm. Prediction with JPred4 (Drozdetskiy et al. 2015) showed MpPAH had an α helix at N-terminus as well as yeast PAH1 and AtPAHs; indicated MpPAH can also interact with membrane via this helix.

We utilized the CRISPR/Cas9 system to carry out genome editing of the first exon of Mp*PAH*, which yielded two knockout lines, named Mp*pah-1* and Mp*pah-2*, in independent trials (Fig. 1b). These lines lacked the same single base in the first exon, which created a stop codon via a frameshift.

**Fig. 2:**
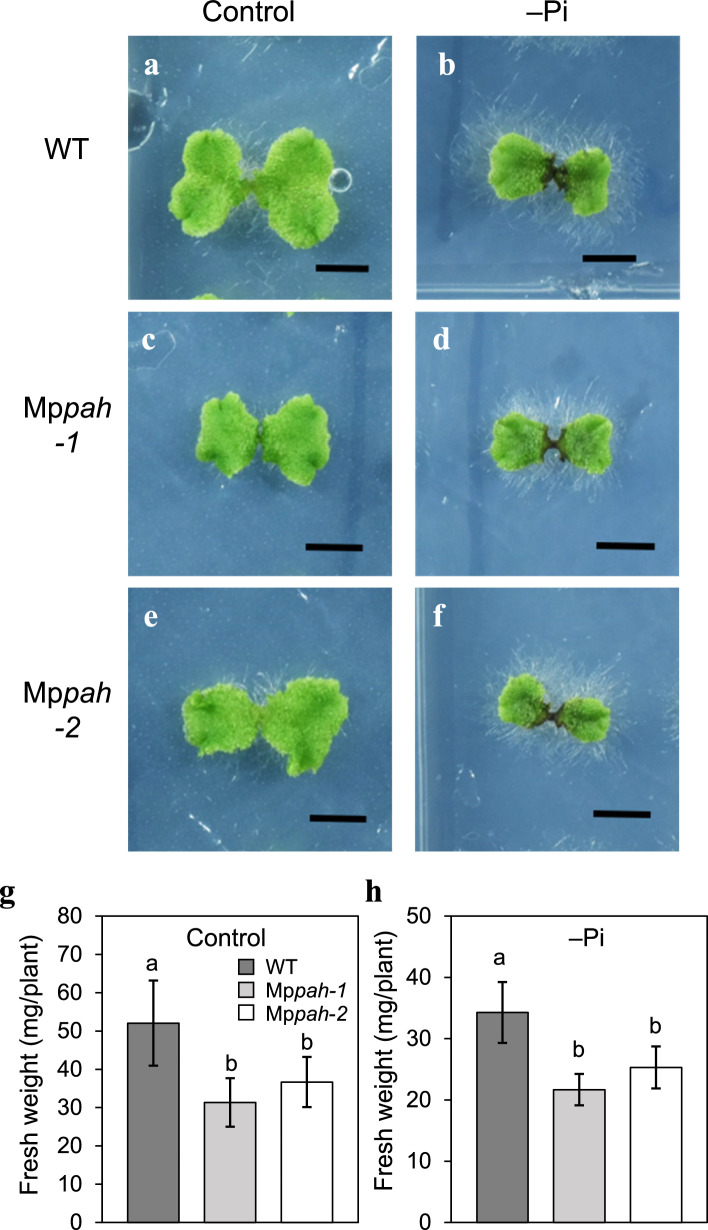
Growth of WT and Mp*pah* mutants cultivated for 6 days under control or –Pi conditions. **a**–**f** WT, Mp*pah-1*, and Mp*pah-2* plants were grown under control conditions (left panels, **a**, **c**, **e**, respectively) or –Pi conditions (right panels, **b**, **d**, **f**). Scale bars, 5 mm. **g** and **h** Fresh weight of WT and Mp*pah* mutants grown under control conditions (**g**) or –Pi conditions (**h**). Values represent the mean ± SD (*n* = 16). Statistical significance was determined with Tukey’s test and denoted by differences in lowercase letters (*P* < 0.01)

An Arabidopsis double knockout mutant of PAH1 and PAH2, called *pah1pah2*, was severely impaired for growth under both –Pi and –N conditions (Nakamura et al. 2009; Yoshitake et al. 2017). To clarify the physiological role of MpPAH in Marchantia under nutrient starvation, we compared the phenotype of WT (*M. polymorpha* strain Tak-1), with the Mp*pah* mutants under nutrient-replete (control) and –Pi conditions (Fig. 2a–f). Although the growth of Mp*pah* mutants was similar to that of WT under control conditions (Fig. 2a, c, e), the fresh weight of the mutant plants was significantly lower (Fig. 2g). Under –Pi conditions, the growth of WT thalli was suppressed whereas the development of rhizoids was enhanced (Fig. 2a, b). However, the rhizoids of Mp*pah* mutants were clearly stunted (Fig. 2b, d, f), and fresh weight was less than that of WT (Fig. 2h). These results suggested that the growth of Marchantia rhizoids under –Pi conditions constituted an easily identifiable phenotype. Comparison of the rhizoid phenotype between WT and Mp*pah* mutants also suggested that MpPAH function is particularly important for rhizoid development regardless of nutrient availability.

### Membrane glycerolipid composition of whole plants is comparable between WT and Mp*pah* mutants even under –Pi conditions

**Fig. 3:**
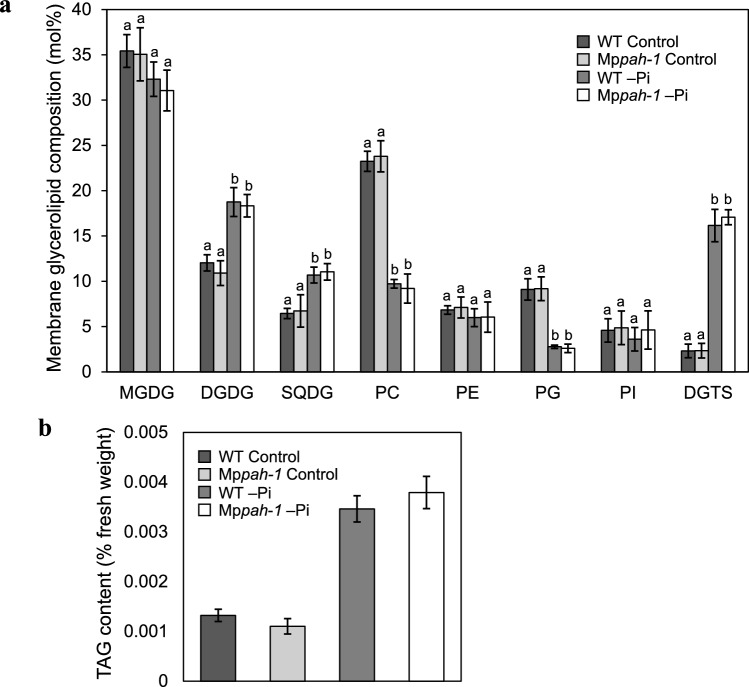
Glycerolipid composition of WT and Mp*pah-1* whole plants grown under controlor –Pi conditions. **a** Composition of membrane glycerolipids of WT and Mp*pah-1* plants grown under control or –Pi conditions. Values represent the mean ± SD (*n* = 4). MGDG, monogalactosyldiacylglycerol; DGDG, digalactosyldiacylglycerol; SQDG, sulfoquinovosyldiacylglycerol; PC, phosphatidylcholine; PE, phosphatidylethanolamine; PG, phosphatidylglycerol; PI, phosphatidylinositol; DGTS; diacylglyceryl-*N,N,N*-trimethylhomoserine. **b** Triacylglycerol (TAG) content of WT and Mp*pah-1* plants grown under control or –Pi conditions. Values represent the mean ± SD (*n* = 3). Statistical significance was determined with Tukey’s test and denoted by differences in lowercase letters (*P* < 0.05)

As the fresh weight of both mutant lines of Mp*pah* was reduced compared with WT, we hypothesized that lipid metabolism was altered and thus growth under –Pi conditions was impaired as shown in Arabidopsis *pah1pah2* double knockout mutant (Nakamura et al. 2009). We analyzed membrane glycerolipid composition using whole plants of Marchantia WT and Mp*pah* grown under control and –Pi conditions (Fig. 3a). In both WT and Mp*pah*, Pi starvation increased the mole percent of DGDG, SQDG and DGTS but decreased that of PC and phosphatidylglycerol among total membrane glycerolipids (Fig. 3a). As for whole plants, membrane glycerolipid composition did not differ significantly between WT and Mp*pah*. The Pi starvation–induced decrease in the mole percent of PC seemed to be compensated by an increase in that of DGDG and DGTS in extra plastidial membranes, suggesting that two types of lipid remodeling, i.e., replacement of phospholipids with galactolipids or with betaine lipids, occur in Marchantia under –Pi conditions. We also analyzed the amount of triacylglycerol in whole plants under –Pi conditions because triacylglycerol was found to accumulate under –Pi conditions in both seed plants and algae (Iwai et al. 2015; Pant et al. 2015; Shimojima et al. 2015). Pi starvation–induced triacylglycerol accumulation was observed in both WT and Mp*pah*, although the amount of triacylglycerol was comparable between them (Fig. 3b). These results demonstrated that the contribution of MpPAH specifically to phospholipid degradation during lipid remodeling under Pi starvation was substantially less than that observed in Arabidopsis (Nakamura et al. 2009).

### Expression of genes involved in lipid remodeling is similar between WT and the Mp*pah* mutants

**Fig. 4:**
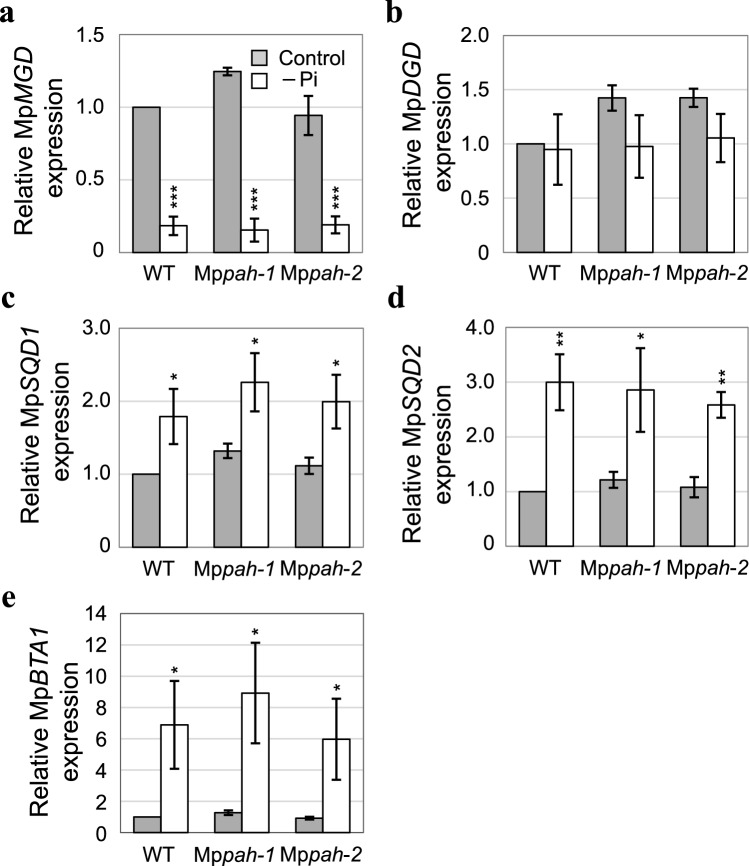
Expression of genes involved in lipid remodeling during Pi starvation. Expression of Mp*MGD* (**a**), Mp*DGD* (**b**), Mp*SQD1* (**c**), Mp*SQD2* (**d**), and Mp*BTA1* (**e**) was normalized to that of Mp*APT*. Values represent the mean ± SD (*n* = 3). Statistical significance was determined with the Student’s *t*-test between control and –Pi conditions for each line. * *P* < 0.05, ** *P* < 0.01, *** *P* < 0.001

Transcriptomic analysis under Pi deficient conditions suggested the expression induction of some Marchantia genes homologous with Arabidopsis lipid turnover-related genes (Rico-Reséndiz et al. 2020). We analyzed how Pi starvation affected the expression of genes involved in lipid remodeling in whole plants of WT, Mp*pah-1*, and Mp*pah-2* (Fig. 4). Five *M. polymorpha* genes, namely Mp*MGD* (Mp2g10950), Mp*DGD* (Mp5g12930), Mp*SQD1* (Mp5g24070) (Rico-Reséndiz et al. 2020), Mp*BTA1* (Mp2g11200) (Murakami et al. 2018), and homologous gene of SQD2 (Fig. S1), Mp*SQD2* (Mp2g10160), were selected. Arabidopsis has three genes encoding MGDG synthases that are classified as type A and type B based on differences in subcellular localization and function (Awai et al. 2001; Kobayashi et al. 2004, 2009). In Arabidopsis plants starved for Pi, the expression of genes encoding type B MGDG synthase is markedly elevated, and the MGDG that is produced is predominantly utilized for DGDG synthesis during lipid remodeling (Kobayashi et al. 2009). Marchantia plants, however, have only one gene for MGDG synthase, i.e., Mp*MGD*, but its expression was rather suppressed under –Pi conditions (Fig. 4a). The fact that expression of Mp*DGD* (encoding DGDG synthase) remained unchanged during Pi starvation (Fig. 4b) suggested that DGDG accumulation under Pi starvation in Marchantia could not be attributed to the upregulation of *MGD* and *DGD*. On the other hand, the expression of each of *SQD1*, *SQD2* and *BTA1* was induced by Pi starvation (Fig. 4c, d, e), which coincided with the increase in the amount of SQDG and DGTS during Pi starvation. These results suggested that, in Marchantia, the Pi starvation–induced accumulation of SQDG and DGTS is regulated at the transcriptional level. Expression of these genes was comparable between WT and Mp*pah* (Fig. 4), indicating that the absence of MpPAH did not substantially affect the expression of other genes involved in lipid remodeling under Pi starvation.

### MpPAH is involved in PC degradation under both control and –Pi conditions

**Fig. 5:**
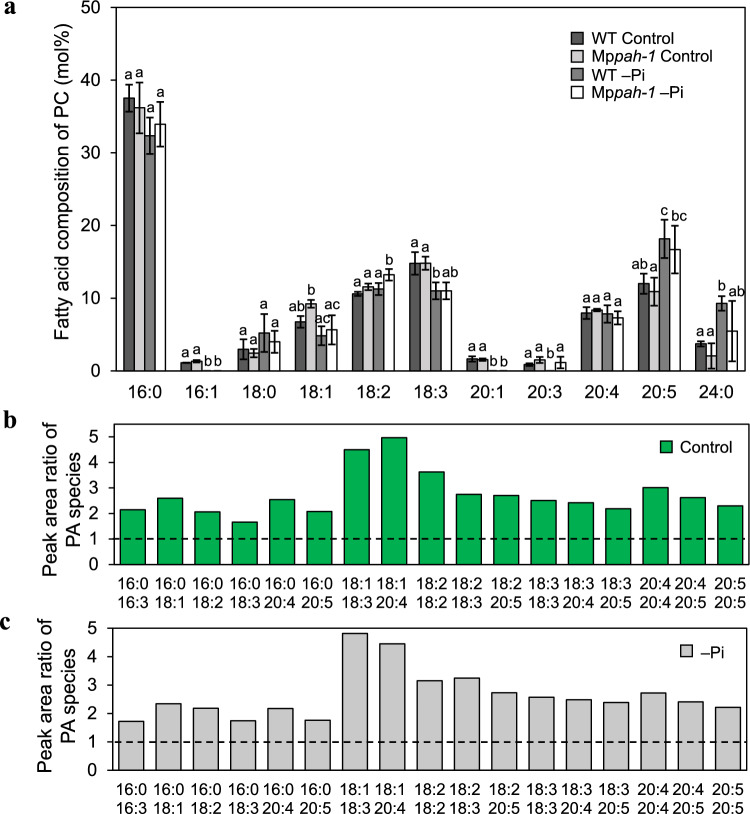
Fatty acid composition of PC and the peak area ratio of PA species in WT and Mp*pah-1* whole plants.** a** Fatty acid composition of PC extracted from WT and Mp*pah-1* whole plants cultivated for 6 days under control or –Pi conditions. Values represent the mean ± SD (*n* = 4). Statistical significance was determined with Tukey’s test and denoted by differences in lowercase letters (*P* < 0.05). **b** and **c** Peak area ratio of PA species of Mp*pah-1* mutant to PA species of WT cultivated for 6 days under control (**b**) or –Pi (**c**) conditions. Values represent the fold-difference in PA species of Mp*pah-1* compared with WT, which was set to 1

The Arabidopsis AtPAHs are considered to dephosphorylate PC-derived PA because the accumulated PC in the *pah1pah2* mutant was found to be highly unsaturated as a feature when PC degradation was suppressed (Nakamura et al. 2009; Eastmond et al. 2010). For the Marchantia Mp*pah* mutants, the mole percent of PC was similar to that of WT regardless of growth condition (Fig. 3a), suggesting that MpPAH contributes little to PC degradation in total membranes. However, the fatty acid composition of PC differed between WT and Mp*pah-1* under both control and –Pi conditions (Fig. 5a). Among the total fatty acids in PC, the mole percent of C18:2 in Mp*pah-1* was slightly higher than that in WT under –Pi conditions, whereas the mole percent of C18:2 was comparable under control conditions (Fig. 5a). Given that PC is desaturated in the ER membrane, PC degradation may have been slightly suppressed in the Mp*pah* mutant under –Pi conditions. Next, we used liquid chromatography coupled with mass spectrometry to analyze PA molecular species among the total lipids extracted from whole plants (Fig. 5b, c). The peak area of each PA species detected in WT was set to 1, with a subsequent comparison with the corresponding area for each PA species in Mp*pah-1*. The ratio of peak areas was greater than 1 regardless of PA species, indicating that the PA content in Mp*pah-1* was greater than that in WT under both control and –Pi conditions. These results confirmed that MpPAH is a de facto PAH and suggested that both de novo synthesized PA and PC-derived PA can serve as substrates.

### The relatively greater amount of 16:3 fatty acids in MGDG in Mp*pah* indicates the involvement of MpPAH in plastid glycolipid synthesis through the ER pathway

**Fig. 6:**
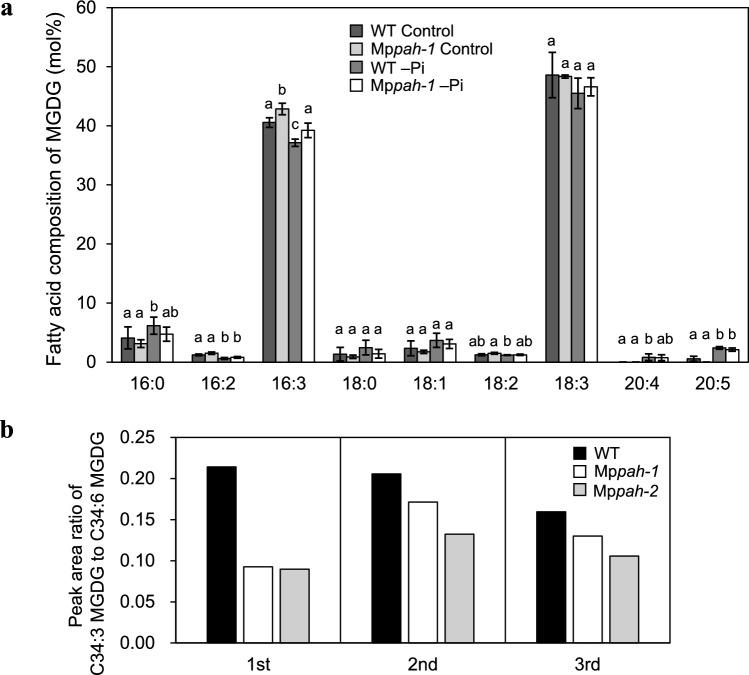
**a** Fatty acid composition of MGDG extracted from WT and Mp*pah-1* whole plants cultivated for 6 days under control or –Pi conditions. Values represent the mean ± SD (*n* = 4). Statistical significance was determined with Tukey’s test and denoted by differences in lowercase letters (*P* < 0.05). **b** Ratio of peak areas of C34:3 MGDG to C34:6 MGDG, as assessed qualitatively in rhizoids of WT, Mp*pah-1*, and Mp*pah-2*. The data denoted as ‘1st’, ‘2nd’ and ‘3rd’ correspond to three independent trials (different samples in each trial) of total lipids extracted from rhizoids of WT and Mp*pah* mutants cultivated for 6 days under control conditions

The fatty acid composition of MGDG provided important information concerning glycolipid synthesis in plastids. MGDG is synthesized via galactose transfer to DAG, which originated either from the ER or via de novo synthesis in plastids (Miège et al. 1999). Most of the MGDG synthesized through the plastid pathway contains C16:3 at the *sn*-2 position, whereas MGDG synthesized through the ER pathway contains no C16 fatty acid at the *sn*-2 position but sometimes has C16:0 at the *sn*-1 position (Ohlrogge and Browse 1995). In Arabidopsis, both the ER pathway and the plastid pathway equally contribute to MGDG synthesis (Browse et al. 1986). As AtPAH is involved in the ER pathway, the ratio of ER-derived to plastid-derived MGDG and the mole percent of MGDG among total membrane glycerolipids were both lower in Arabidopsis *pah1pah2* compared with those in WT (Nakamura et al. 2009; Eastmond et al. 2010). However, the mole percent of MGDG among total membrane glycerolipids was comparable between WT and Mp*pah* under both control and –Pi conditions (Fig. 3a). To investigate the involvement of MpPAH in the ER pathway, we compared the fatty acid composition of MGDG between WT and Mp*pah* (Fig. 6a). In WT plants grown under control conditions, the relative amount of C16:0 to C16:3 was quite low (~ 0.1), which clearly indicated that ~ 90% of the MGDG is synthesized through the plastid pathway in Marchantia (Fig. 6a); this was consistent with results reported by Hirashima et al. (2021). In WT plants grown under –Pi conditions, however, the mole percent of C16:3 in MGDG was lower whereas that of C16:0 was higher when compared with those values measured under control conditions (Fig. 6a). These results indicated that, compared with control conditions, MGDG synthesis during Pi starvation is more dependent on the ER pathway. In Mp*pah*, the mole percent of C16:3 in MGDG was greater than that in WT under both control and –Pi conditions, whereas that of C16:0 in MGDG was comparable between WT and Mp*pah* (Fig. 6a). These results clearly showed that MpPAH contributes to plastid glycolipid synthesis through the ER pathway under both control and –Pi conditions.

In Arabidopsis, the contribution of the ER pathway to glycolipid synthesis in plastids is greater in root cells and guard cells than in leaf mesophyll cells (Awai et al. 2001; Kobayashi et al. 2009; Negi et al. 2018; Obata et al. 2021), suggesting that the degree of dependence on the ER pathway varies among plant tissues and cell types. The Marchantia rhizoid is a macroscopic single cell that develops from the ventral epidermis, and rhizoids constitute a distinct cell type in Marchantia (Shimamura 2016). Because rhizoid elongation was inhibited in Mp*pah* (Fig. 2c–f), we also analyzed the amount of C34 MGDG in rhizoids (Fig. 6b). Total glycerolipids in rhizoids were extracted from WT, Mp*pah-1* and Mp*pah-2* plants grown under control conditions. C34:3 (C16:0, C18:3) MGDG synthesized through the ER pathway and C34:6 (C16:3, C18:3) MGDGs synthesized through the plastid pathway were detected qualitatively with liquid chromatography–coupled mass spectrometry. The ratio of peak areas for C34:3 MGDG to C34:6 MGDG in Mp*pah* was smaller than that in WT in each of the three independent trials. These results confirmed that MpPAH contributes to MGDG synthesis through the ER pathway in rhizoids as well. Because ER-derived MGDG is a major substrate for DGDG synthesis in Arabidopsis owing to the substrate specificity of DGDG synthase (Browse et al. 1986), plastid glycolipids such as MGDG and DGDG produced through the ER pathway might play an important role in rhizoid elongation, which is essential for Marchantia growth.

### Mp*pah* accumulates PC during N starvation, and Mp*pah* growth is sensitive to both N and Pi starvation

**Fig. 7:**
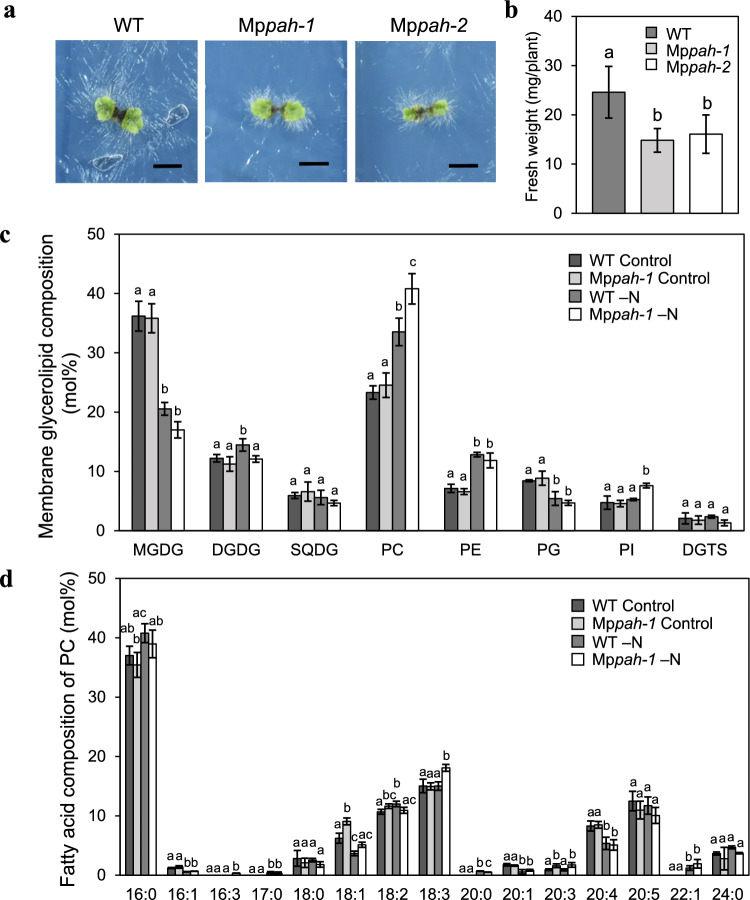
Comparison of WT and Mp*pah* mutants cultivated for 6 days under –N conditions. **a** Growth of WT and Mp*pah-1* and Mp*pah-2* under –N conditions. Scale bars, 5 mm. **b** Fresh weight of WT and Mp*pah* mutants under –N conditions. Values represent the mean ± SD (*n* = 16). Statistical significance was determined with Tukey’s test and denoted by differences in lowercase letters (*P* < 0.01). **c** and **d** Glycerolipid composition (**c**) and fatty acid composition of PC (**d**) of WT and Mp*pah-1* whole plants grown under control or –N conditions. Values represent the mean ± SD (*n* = 4 each). PE, phosphatidylethanolamine; PG, phosphatidylglycerol; PI, phosphatidylinositol.Statistical significance in **c** and **d** was determined with Tukey’s test and denoted by differences in lowercase letters (*P* < 0.05)

The growth of Arabidopsis *pah1pah2* mutants is suppressed under both –N and –Pi conditions (Nakamura et al. 2009; Yoshitake et al. 2017). Thus, we also examined the impact of N starvation on Marchantia growth (Fig. 7). Under –N conditions, WT Marchantia thalli grew very little, and thalli ultimately became pale green; however, rhizoid elongation was enhanced, as was observed under –Pi conditions (Fig. 7a, Fig. 2b). Under –N conditions, the growth suppression of thalli was more pronounced in Mp*pah* than WT (Fig. 7a), and fresh weight of Mp*pah* was significantly lower than that of WT (Fig. 7b). In WT during N starvation, the mole percent of MGDG and of phosphatidylglycerol decreased whereas that of DGDG, PC and phosphatidylethanolamine increased among total membrane glycerolipids (Fig. 7c), which was similar to what was reported for Arabidopsis WT plants (Gaude et al. 2007; Yoshitake et al. 2017). In contrast, for Mp*pah* mutants subjected to N starvation, the mole percent of PC increased more than in WT, whereas the mole percent of DGDG remained unchanged (Fig. 7c). We found that the fatty acid composition of MGDG under control or –N conditions did not differ significantly between WT and Mp*pah* plants (Fig. S2). However, the composition of their membrane glycerolipids differed significantly in terms of mole percent of DGDG and PC specifically under –N conditions (Fig. 7c). Under –N conditions, the mole percent of PC in Mp*pah* was greater than that of WT (Fig. 7c), suggesting that MpPAH is involved in the degradation of PC. Under –N conditions, the fatty acid composition data for PC in Mp*pah* revealed a higher mole percent of C18:3 compared with WT (Fig. 7d), suggesting that PC degradation was slightly suppressed in Mp*pah* specifically under –N conditions. These results suggested that the predominant substrate for MpPAH is PC-derived PA—at least during N starvation. On the other hand, the mole percent of DGDG in Mp*pah* was lower than that in WT under –N conditions (Fig. 7c). Given that the mole percent of C16:3 constituted ~ 15% of the total fatty acids in DGDG, we estimated that ~ 30% of DGDG is derived from MGDG synthesized through the plastid pathway and ~ 70% through the ER pathway (Fig. S3). Although the fatty acid composition of DGDG was comparable between WT and Mp*pah* under both control and –N conditions (Fig. S3), the lower mole percent of DGDG in Mp*pah* might be a consequence of the suppression of plastid glycolipid synthesis through the ER pathway.

**Fig. 8:**
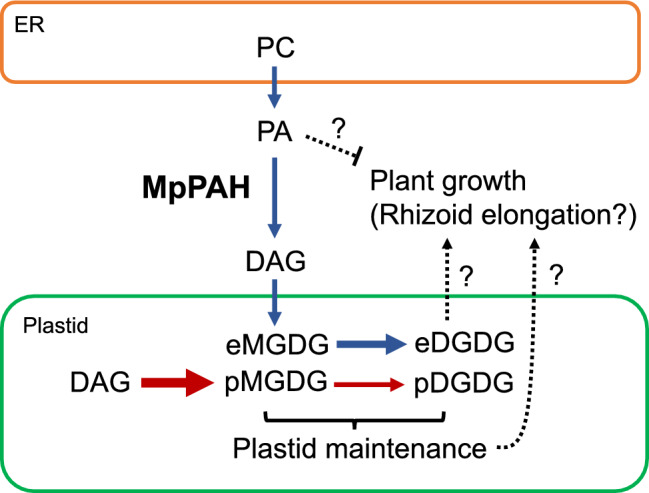
Model depicting lipid flux in Marchantia thallus cells. In Marchantia, MGDG is predominantly synthesized through the plastid pathway (red arrows; denoted as pMGDG). MpPAH is a phosphatidic acid phosphohydrolase for which the substrate is PA derived from PC and is involved in MGDG synthesis through the ER pathway (blue arrows; denoted as eMGDG). Approximately 70% of DGDG is synthesized through the ER pathway (denoted as eDGDG). Rhizoid elongation might be regulated by local intracellular concentrations of PA and eDGDG (as a constituent of extraplastidial membranes and for plastid maintenance). Dashed arrows indicate plausible effects. pDGDG denotes DGDG synthesized through the plastid pathway. *PC* phosphatidylcholine, *PA* phosphatidic acid, *DAG* diacylglycerol, *MGDG* monogalactosyldiacylglycerol, *DGDG* digalactosyldiacylglycerol

In total, these results suggested that MpPAH has a significant role during Marchantia growth even under nutrient-replete conditions but that MpPAH contributes also to survival under –Pi and –N conditions. The substrate for MpPAH is PC-derived PA and the DAG that is produced as a substrate for plastid glycolipid synthesis through the ER pathway (Fig. 8).

## Discussion

In this study, we found that the absence of Mp*PAH* resulted in a decrease in fresh weight with shorter rhizoids compared to WT even under nutrient-replete conditions. Although the mole percent of each glycerolipid among total membrane lipids from the whole plant was not significantly affected in Mp*pah* under nutrient-replete conditions, the fatty acid composition of MGDG indicated that the amount of plastid glycolipids produced through the ER pathway was suppressed. Mp*pah* also accumulated PA both under nutrient-replete and nutrient-starved conditions, but PC accumulation was not observed under nutrient-replete and Pi-starved conditions. In Arabidopsis, the PAH-deficient double mutant *pah1pah2* has been reported to accumulate PC and PA even under nutrient-replete growth conditions, but growth did not differ significantly between WT and the mutant under nutrient-replete conditions (Nakamura et al. 2009; Eastmond et al. 2010). The growth of *pah1pah2* plants was markedly suppressed under Pi or N starvation, with a concomitant decrease in the amount of galactolipids relative to phospholipids (Nakamura et al. 2009; Yoshitake et al. 2017). Under Pi starvation, the absence of AtPAH in *pah1pah2* plants promoted PC synthesis in the ER and reduced the supply of DAG from the ER to plastids (Nakamura et al. 2009; Eastmond et al. 2010). A decrease in the amount of Pi released by PC degradation clearly explained the negative effect on the growth of the mutant under Pi starvation. In contrast, under N starvation, AtPAH was reported to contribute to the maintenance of the chloroplast membrane and its function by increasing the supply of DAG for MGDG synthesis (Yoshitake et al. 2017). In Marchantia, however, the composition of membrane lipids in whole plants of the PAH-deficient Marchantia mutant Mp*pah* did not differ significantly from that of WT under both nutrient-replete and Pi-deficient conditions. Nevertheless, the growth of Mp*pah* plants was suppressed not only under Pi and N starvation but also under nutrient-replete conditions. Surprisingly, phospholipid degradation during lipid remodeling was not suppressed in Mp*pah*, clearly indicating that PAH is not the predominant enzyme that degrades phospholipids during Pi starvation in Marchantia and suggesting that other unknown phospholipases are involved in phospholipid degradation under Pi starvation. Analysis of the fatty acid composition of Marchantia MGDG revealed that MGDG synthesis in WT Marchantia is dependent on the plastid pathway. In other words, even if the ER pathway is suppressed by PAH deficiency, sufficient amounts of MGDG and DGDG can be synthesized through the plastid pathway.

In the present study, lipid fractions extracted from whole plants of Marchantia WT and Mp*pah* were used for the analysis of membrane glycerolipid composition, which can negate the effects of PAH deficiency in microtissues. Comparing the growth of WT and Mp*pah* plants revealed a tendency for differences in the elongation of rhizoids, suggesting that rhizoid development might have been perturbed by the absence of PAH. PAH in Marchantia may contribute to the growth through the control of lipid composition locally rather than in the whole plant. In Arabidopsis, mesophyll cells and roots differ in membrane glycerolipid composition and in their dependence on the two pathways for glycolipid synthesis in plastids, namely the plastid pathway and the ER pathway (Obata et al. 2021). In Arabidopsis, the synthesis of plastid glycolipids in leaves is equally dependent on the plastid pathway and the ER pathway, whereas that in roots is much more dependent on the ER pathway. The degree of dependence can be estimated from the percentage of C16:3 fatty acids in MGDG. In Arabidopsis roots, MGDG comprises ~ 5% (molar basis) of total membrane glycerolipids, but this MGDG contains almost no C16:3 under nutrient-replete or Pi-deficient conditions. This indicates that the synthesis of plastid glycolipids in roots depends on DAG that is synthesized through the ER pathway (Kobayashi et al. 2009). In addition, in Arabidopsis, glycolipid synthesis in plastid pathway is severely downregulated in guard cells (Negi et al. 2018). Because the rhizoid of liverworts is also a single cell derived from epidermal cells and contains plastids, it is possible that plastid lipids may be mostly synthesized through the ER pathway and thus greatly affected when PAH is deficient. However, the correlation, if any, between the synthesis of plastid glycolipids and root development has not been demonstrated. One hypothesis is that DGDG is a component of extra plastidial membranes such as the ER, mitochondria, and the plasma membrane (Härtel et al. 2000; Jouhet et al. 2004; Andersson et al. 2003, 2005). In Arabidopsis, DGDG is predominantly produced from ER-derived MGDG (Browse et al. 1986; Kunst et al. 1988); likewise, in Marchantia, ~ 70% of DGDG is synthesized from ER-derived MGDG (Fig. S3). In seed plants, MGDG synthesis through the ER pathway is upregulated during Pi starvation, and the DGDG that is produced is exported to extra plastidial membranes to maintain plant growth (Härtel et al. 2000; Andersson et al. 2003). Oversupply of sucrose in the growth medium enhances plant growth, with upregulation of type B MGDG synthesis and, subsequently, DGDG synthesis (Murakawa et al. 2014). Arabidopsis WT plants starved for N could not upregulate type B MGDG synthases, whereas overexpression of PAH1 or PAH2 led to an increase in the cellular abundance of type B MGDG synthases, resulting in better growth than WT (Yoshitake et al. 2017). Thus, in the case of Arabidopsis, MGDG synthesis through the ER pathway can be upregulated in response to an abiotic stress to maintain plant growth by upregulating the expression of genes encoding type B MGDG synthases. In Marchantia, the absence of presumed lipid transporter STAR2, which is involved in the supply of ER-derived C20 fatty acids to plastid glycolipids, results in decreased fresh weight under Pi deprivation (Hirashima et al. 2021). Our observed decrease in the fresh weight of whole plants of Mp*pah* might be a consequence of even slight suppression of plastid glycolipid synthesis through the ER. The rhizoid functions to attach the thallus to the soil and to help the thallus efficiently absorb water and inorganic ions (Jones and Dolan 2012; Cao et al. 2014). We grew thalli on a cellophane sheet to avoid them from being embedded in the medium. Impaired Mp*pah* rhizoid development might affect formation of optimal contact area to uptake materials from the flat surface of a medium. It is plausible that normal growth was impaired in not only rhizoids but also the thallus in Mp*pah* because the development of certain organs, such as the rhizoid, is more dependent on the ER pathway. It is also possible that the growth suppression observed in Mp*pah* may be a consequence of PA accumulation. PA is known as a signaling molecule accumulated in response to various stress, and its generation is controlled by phospholipase D and diacylglycerol kinase. Phospholipase D hydrolyzes phospholipids such as PC and PE to produce PA, whereas diacylglycerol kinase phosphorylates DAG (Testerink and Munnik 2005). In particular, phospholipase D ζ and ε contribute to maintain growth under phosphate and nitrogen starvation, respectively (Cruz-Ramírez et al. 2006; Li et al. 2006; Hong et al. 2009). The alteration of PA level observed in Mp*pah* might have been the cause of growth suppression as a stress-response strategy.

Suppression of rhizoid elongation in Marchantia suggested that the effect of PAH knockout on the glycerolipid composition of rhizoids needs to be investigated. The rhizoid cell elongates by tip growth, the mechanism of which partially overlaps with that of Arabidopsis (Honkanen et al. 2016; Proust et al. 2016; Otani et al. 2018). Tip growth consumes lipids as the component of the plasma membrane. During the tip growth of the pollen tube, numerous secretory vesicles deliver cell wall materials from the Golgi apparatus to the apex, but only a part of membrane is estimated to be recovered via endocytosis (Derksen et al. 1995). Not only phospholipids but also glycolipid synthesis is indicated to be involved in tip growth in Arabidopsis pollen tube (Kobayashi et al. 2004). In our present study, however, it was difficult to isolate and collect a sufficient amount of rhizoids for lipid analysis without contaminating the sample with other tissues. Therefore, in our analysis of rhizoid lipids (Fig. 6b), only a small amount of highly pure rhizoid tissue could be collected, and liquid chromatography–coupled mass spectrometry was used instead of gas chromatography coupled with a flame ionization detector, which is conventionally considered to be the most quantitative as long as it compares the peak area ratio of two molecular species. The results revealed that MGDG synthesis through the ER pathway was suppressed in Mp*pah* even in the rhizoid. However, it was not possible to carry out a detailed investigation of whether Mp*PAH* knockout affected the abundance of membrane glycerolipids, including MGDG, in rhizoids. Thus, the quantification of each glycerolipid species in the rhizoid would shed light on the physiological role of PAHs in liverworts.

### Supplementary Information

Below is the link to the electronic supplementary material.

Supplementary Fig. S1 Phylogenetic tree of SQD2. Fig. S2 Fatty acid composition of MGDG of WT and Mppah-1 cultivated for 6 days under control or –N conditions. Fig. S3 Fatty acid composition of DGDG under nutrient-starved conditions. file1 (PDF 315 KB)

## Data Availability

All data generated or analyzed during this study are included in this published article and its supplementary information files.

## Abbreviations

DAG: Diacylglycerol
DGDG: Digalactosyldiacylglycerol
DGTS: Diacylglyceryl-*N,N,N*-trimethylhomoserine
MGDG: Monogalactosyldiacylglycerol
PA: Phosphatidic acid
PAH: Phosphatidic acid phosphohydrolase
PC: Phosphatidylcholine
SQDG: Sulfoquinovosyldiacylglycerol
