# Supplementary material for: Phosphatidic acid phosphohydrolase modulates glycerolipid synthesis in Marchantia polymorpha and is crucial for growth under both nutrient-replete and -deficient conditions
Source: Planta. 2023 Oct 4;258(5):92. doi: 10.1007/s00425-023-04247-4 (PMC10550880; doi:10.1007/s00425-023-04247-4)
Supplement: Supplementary file 1 — Supplementary Fig. S1 Phylogenetic tree of SQD2. Fig. S2 Fatty acid composition of MGDG of WT and Mppah-1 cultivated for 6 days under control or –N conditions. Fig. S3 Fatty acid composition of DGDG under nutrient-starved conditions. file1 (PDF 315 KB) [file 425_2023_4247_MOESM1_ESM.pdf]

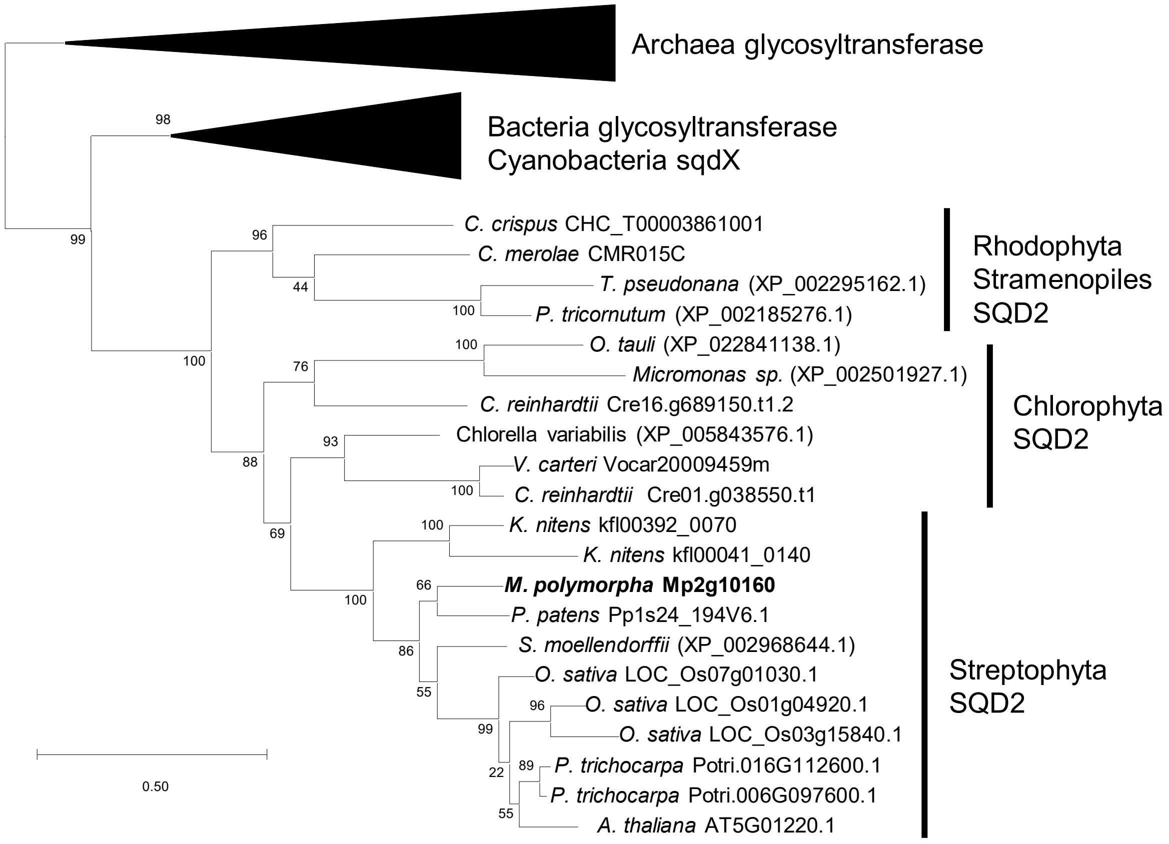

Fig. S1 Phylogenetic tree of SQD2. Phylogenetic analysis using maximum likelihood was performed with MEGA7 (the ML heuristic method: the Subtree–Pruning–Regrafting (SPR) algorithm with search level 5; a branch swap filter: Very Strong; The amino acid substitution model: LG with 8 gamma categories). Bootstrap values represent data for 500 replicates. The tree was drawn to scale, with each branch lengths indicating the number of substitutions per site.

Fig. S1 Shimojo et al.

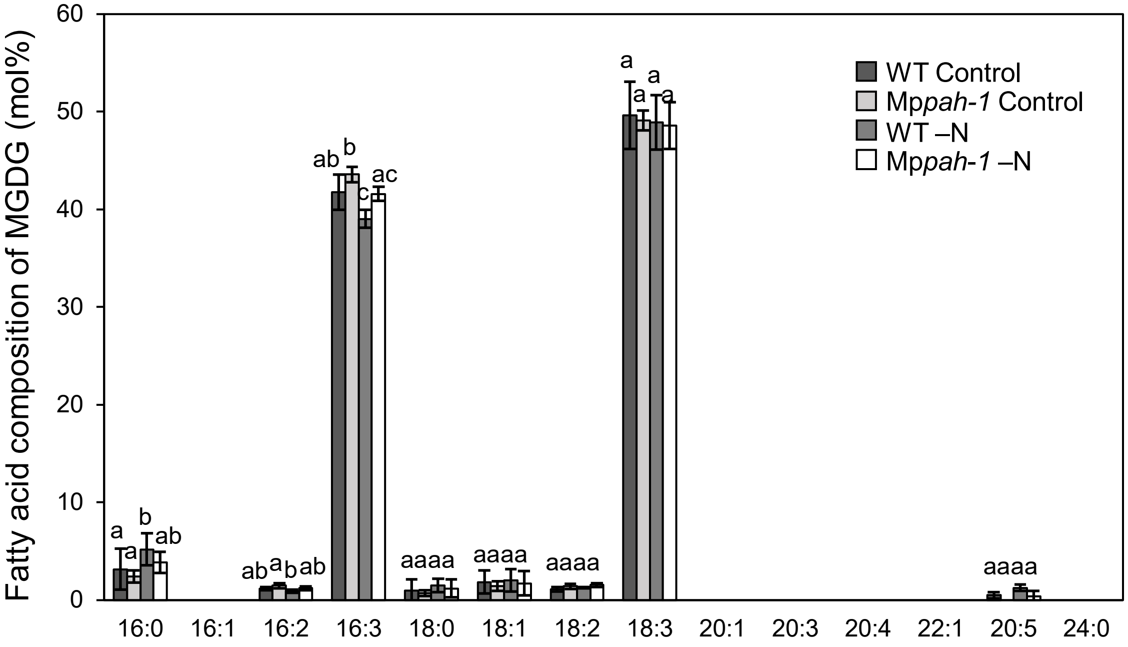

Fig. S2 Fatty acid composition of MGDG of WT and Mp*pah-1* cultivated for 6 days under control or –N conditions. Values were the mean ± SD ($n = 4$). Statistical significance was determined with Turkey's test and denoted by letters at the top ($P<0.05$).

Fig. S2 Shimojo et al.

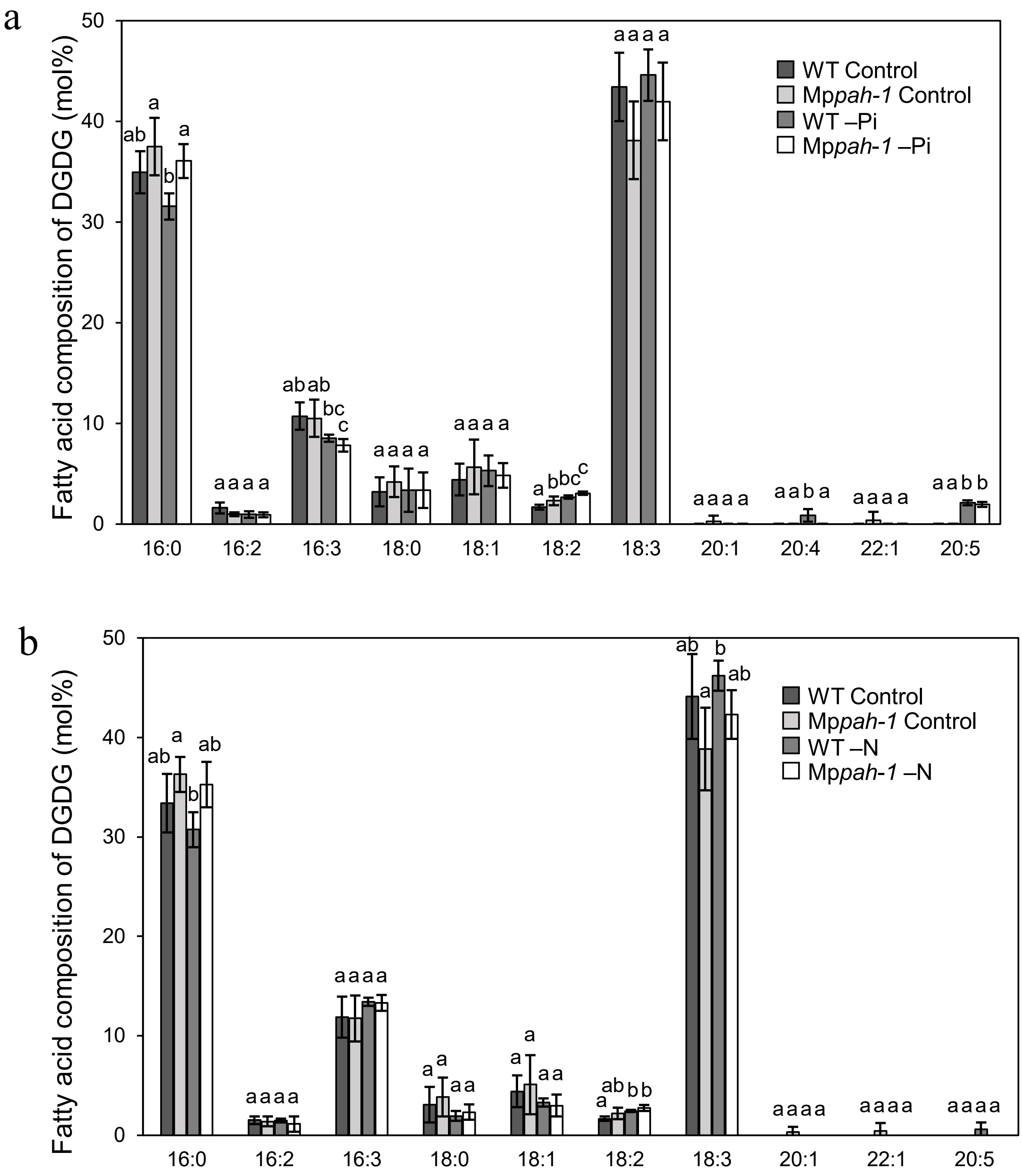

Fig. S3 Fatty acid composition of DGDG under nutrient-starved conditions. a, the comparison of WT and Mp*pah-1* cultivated 6 days under the control or –Pi condition. b, the comparison of WT and Mp*pah-1* cultivated 6 days under the control or –N condition. Values were the mean ± SD (n = 4). Statistical significance was determined with Turkey's test and denoted by letters at the top (P<0.05).

Fig. S3 Shimojo et al.
